# Quality of Life for Patients with Down Syndrome and Their Caregivers: A Cross-Sectional Study from a Parental Perspective in Saudi Arabia

**DOI:** 10.3390/healthcare13131614

**Published:** 2025-07-06

**Authors:** Amal Khaleel AbuAlhommos, Maitham Abdullah Al Hawaj, Ashwaq Ali Alanazi, Hanadi Hwthael Alrashidi, Maha Faleh Aldawsari, Rasan Ali Alajmi

**Affiliations:** Department of Pharmacy Practice, College of Clinical Pharmacy, King Faisal University, Al Ahsa 31982, Saudi Arabia; hawaj@kfu.edu.sa (M.A.A.H.); ashwaqalianezi@gmail.com (A.A.A.); 220031648@student.kfu.edu.sa (H.H.A.); 220005880@student.kfu.edu.sa (M.F.A.); 220019412@student.kfu.edu.sa (R.A.A.)

**Keywords:** caregivers, children, Down syndrome, parents, quality of life

## Abstract

Background: Patients with Down syndrome (DS) commonly experience psychological and mental problems. Studying the quality of life (QoL) of children with DS is important because it increases knowledge related to understanding the challenges that this group may face. This study aims to examine the QoL of children with DS from a parental perspective in terms of physical, emotional, social, and school domains, depending on several factors, and identify demographic characteristics of their parents that may affect their QoL. Methods: This online survey study was conducted in Saudi Arabia between November 2024 and March 2025. The inclusion criteria targeted parents of children with confirmed DS diagnoses aged between 8 and 18 years. Results: The findings of this study showed that children with DS aged between 0 and 2 years had significantly lower QoL scores (10.18 ± 3.83) compared to other age groups (*p* = 0.02). In addition, gender differences were significant in the emotional (*p* = 0.03), social (*p* = 0.01), and school (*p* = 0.01) domains, with females scoring lower QoL scores in all areas compared to males. Moreover, educational level showed significant results across all domains, particularly for children with no education, who had the lowest QoL scores in the physical domain (22.34 ± 7.53, *p* = 0.004), emotional domain (10.41 ± 3.79, *p* = 0.003), social domain (11.22 ± 4.06, *p* = 0.001), and school domain (8.75 ± 5.09, *p* = 0.001). The findings of this study showed that children with DS who are in primary school (odds ratio (OR) = 5.90, 95% confidence interval (CI): 1.85–18.78, *p* = 0.003) and middle school (OR = 5.27, 95% CI: 1.44–19.31, *p* = 0.012) had significantly higher odds of better QoL compared to children with no formal education. Additionally, children cared for by their fathers had significantly lower odds compared to those cared for by their mothers (OR = 0.07, 95% CI: 0.01–0.90, *p* = 0.041). None of the demographic characteristics of caregivers reached a statistical significance level to have influence on caregivers QoL (*p* > 0.05). Conclusions: The findings of this study demonstrated a low level of QoL, affecting the emotional, social, and school domains, especially among female children with DS aged between 0 and 2 years with no formal education and cared for by their fathers. Governments should develop a comprehensive plan to care for these children and families in order to enhance their rights and quality of life, thereby placing emphasis on those who exhibit parameters related to a lower QoL.

## 1. Introduction

Down syndrome (DS) is a genetic disorder that causes intellectual disability due to an additional chromosome 21 [1]. People with this syndrome experience symptoms that affect their body systems, including the muscles, bones, nerves, and heart. A person with DS can be distinguished by several external characteristics, including short stature, hypotonia, delayed mental abilities, and heart problems. DS makes the affected individual more susceptible to immune, glandular, blood, hearing, and vision problems. Patients with DS also experience psychological and mental problems [2]. The main cause of DS is an additional chromosome 21, which leads to characteristic dysmorphic features and affects the brain and cognitive abilities. This mutation occurs due to chromosomes not separating properly or due to the lack of meiosis [3,4]. The number of cases of DS recorded in Saudi Arabia from 1982 to 1991 was one in every 554 births [5]. In 2015, a study published in Saudi Arabia revealed that 6.6 cases of DS were recorded in every 10,000 children from 2004 to 2005 [6]. Improving the quality of life (QoL) among children with DS is important for their emotional, behavioral, and physical health. A number of studies have emerged that confirm the importance of involving this group in various activities that enable them to improve their growth and behavior. However, in this context, it must be noted that there are few studies related to this topic [7]. Based on the general concept established by the World Health Organization (WHO), the QoL for children with DS is the ability to meet their needs due to their limited capabilities, whether physical, emotional, or social. The WHO emphasizes the importance of community cooperation with this group to carry out activities that help them to integrate into society and their self-reliance [8]. Studying the QoL of children with DS is extremely important because it increases knowledge related to understanding the challenges that this group may go through, whether social, mental, or physical, in addition to identifying the health problems that accompany them, and most importantly, supporting the families of these children in order to help them to enjoy a good standard of life. This study aims to examine the QoL of children with DS from a parental perspective in terms of physical, emotional, social, and school domains, depending on several factors, and identify demographic characteristics of their parents that may affect their QoL. The findings of this study provide insights on the national level related to the influence of social norms and the healthcare system on the QoL of DS patients and their caregivers. This facilitates comparisons across other studies globally and identifies any cultural differences.

## 2. Methods

### 2.1. Study Design

This is an online survey study conducted in Saudi Arabia between November 2024 and March 2025.

#### 2.1.1. Study Population and Recruitment Procedure

The inclusion criteria targeted parents of children with confirmed DS diagnoses aged between 8 and 18 years with or without medications. The exclusion criteria ruled out parents of children with DS over the age of 19 and individuals from the general population. The study participants were recruited using social media platforms (X, Snapchat, and Facebook). The survey link was distributed across these social media platforms and asked participants who met the inclusion criteria to participate. The convenience sampling technique was employed to recruit the study participants who met the inclusion criteria and showed interest in participating in this study. The inclusion criteria were highlighted in the study invitation letter.

The questionnaire tools were self-administered and completed by caregivers and legal guardians for people with DS in parallel.

#### 2.1.2. Study Instruments

We evaluated the QoL of children with DS using the Pediatric Quality of Life Inventory (PedsQL) [9]. The PedsQL is a brief measure of QoL in children. This instrument can be completed by parents and children. The 23 items in the PedsQL comprise four generic core scales: physical functioning (8 items), emotional functioning (5 items), social functioning (5 items), and school functioning (5 items). Items on the PedsQL Scales are reverse scored and transformed to a 0–100 scale. Higher scores indicate better QoL (0 (“Never”) = 100, 1 (“Almost Never”) = 75, 2 (“Sometimes”) = 50, 3 (“Often”) = 25, and 4 (“Almost Always”) = 0) [9]. The QoL for caregivers was evaluated using the World Health Organization Quality of Life (WHOQOL)-BREF [8]. It is a 26-item questionnaire instrument that produces scores for four domains related to QoL: physical health, psychological health, social relationships, and environment. Each item is rated on a 5-point Likert scale, reflecting the person’s experiences over the preceding two weeks.

### 2.2. Ethical Approval

Ethical approval for this study was obtained from the Research Ethics Committee at King Faisal University (Ref. No. KFU-REC-2024-NOV-ETHICS2832). Informed consent was obtained from all caregivers and legal guardians of people with DS.

### 2.3. Data Analysis

The data were analyzed using the Statistical Package for the Social Sciences (SPSS, version 29). Categorical variables, such as participants’ demographic characteristics, were expressed as frequency and percentage, while continuous data, such as scores, were reported as mean and standard deviations (SD), as the data were distributed normally. Normality was checked using histogram, skewness, and kurtosis measures. The QoL for children with DS was categorized into four domains: physical, emotional, social, and school. Each one consists of multiple items rated on a 5-point Likert scale. The total score was calculated by summing the item scores, and the final score was obtained by averaging the total sum. The median total score was used as the cutoff point for multiple logistic regression (the dependent variable). Demographic characteristics were included as independent variables in the regression model. The findings of the regression analysis were presented as the adjusted odds ratio (AOR) with its corresponding 95% confidence interval. The Kolmogorov–Smirnov test was used to assess normality. Since the data met parametric assumptions, the Analysis of Variance (ANOVA) and the independent *t*-test were conducted when applicable. For multiple group comparisons, the Tukey post hoc test was applied, and the results were evaluated accordingly. A similar analysis was performed for the QoL score of caregivers, with the exception that a transformation equation was applied for each score of the WHOQOL-BREF scale, as shown below. The level of significance was defined as a *p*-value less than 0.05.

$$Transformtion\;Scale=\frac{\left(Actual\;raw\;score-Lowest\;possible\;raw\;score\right)}{Possible\;raw\;score\;range}\times 100$$

## 3. Results

### 3.1. Patients’ Perspectives

The analysis included 229 children with DS. The majority were aged 3–11 years (79, 34.5%). There were 126 females (55%). Regarding residency, most children lived in the East (91, 39.7%).

In terms of education, 77 children (33.6%) were in primary school. The majority of children (165, 72.1%) had parents who were not working in the health sector. Regarding caregiving, 157 children (70.7%) were cared for by both parents, Table 1.

The table below presents the challenges faced by children with DS in the physical, emotional, social, and school-related domains based on the perspective of their parents. In terms of physical challenge, 43 children (18.8%) reported never having a problem with walking long distances. With running, 32 (14%) never had a problem. Regarding sports activities, 32 (14%) never had a problem. In carrying heavy objects, 30 (13.1%) never had a problem. As for emotional issues, 68 (29.7%) never felt afraid. Social challenges were also noted, with 79 (34.5%) never experiencing mockery from peers. Regarding school-related difficulties, 62 (27.1%) never had trouble concentrating. These results reflect the range of difficulties children with DS face across various aspects of life, mainly for physical tasks, Table 2.

In the emotional domain, children aged 0–2 years had significantly lower QoL scores (10.18 ± 3.83) compared to other age groups such as 15–18 years old (12.52 ± 4.24) (*p* = 0.02), while in the school domain, children in the 0–2 years age group also had significantly lower QoL scores (9.62 ± 5.31) compared to 15–18 years old (12.78 ± 4.93) (*p* = 0.004). Gender differences were significant in the emotional (*p* = 0.03), social (*p* = 0.01), and school (*p* = 0.01) domains, with females scoring lower QoL scores in all areas compared to males. Educational level showed significant results across all domains, particularly for children with no education, who had the lowest QoL scores in physical (22.34 ± 7.53, *p* = 0.004), emotional (10.41 ± 3.79, *p* = 0.003), social (11.22 ± 4.06, *p* = 0.001), and school (8.75 ± 5.09, *p* = 0.001) domains, which reflects demographic disparities in participants’ QoL and well-being. Further details about the QoL score across the children’s demographics are provided in Table 3.

### 3.2. Caregivers’ Profile

This study included 171 participants, with the highest proportion aged 19–25 (68, 39.8%). The majority were females (132, 77.2%). Regarding high school, 37 (21.6%) had a middle school education. Most participants (156, 91.2%) did not have a parent working in the healthcare sector. Additional details about the demographic characteristics of participants are provided in Table 4.

The majority of participants rated their QoL positively, with 78 (45.6%) considering it good. Regarding health satisfaction, 71 (41.5%) were satisfied. Enjoyment of life varied, with 58 (33.9%) rating it moderate. A significant number (52, 30.4%) felt their lives were meaningful. Sleep satisfaction was moderate among 58 (33.9%). Additionally, 57 (33.3%) were satisfied with themselves. Negative feelings, such as anxiety or depression, were experienced sometimes by 55 (32.2%), Table 5.

In the physical domain, caregivers with an income above 20,000 reported the highest mean score (66.27 ± 16.40, *p* = 0.04), while those with no formal education had the lowest (40.71 ± 10.29, *p* = 0.001). Residency also showed significant differences, with caregivers from the East reporting the highest mean score (59.97 ± 18.20, *p* = 0.001). In the social relationship domain, the education level significantly influenced scores (*p* = 0.05), with diploma holders scoring the highest (73.61 ± 22.62), Table 6.

### 3.3. Factors Affecting the QoL of Patients and Their Caregivers

Table 7 shows factors affecting DS children’s QoL, children in primary school (OR = 5.90, 95% CI: 1.85–18.78, *p* = 0.003) and middle school (OR = 5.27, 95% CI: 1.44–19.31, *p* = 0.012) had significantly higher odds of better QoL compared to children with no formal education. Additionally, children cared for by their fathers had significantly lower odds compared to those cared for by their mothers (OR = 0.07, 95% CI: 0.01–0.90, *p* = 0.041). Further details about the factors associated with QoL in children with DS are provided in Table 7.

However, Table 8 shows that none of these factors reached a statistical significance level to have an influence on caregivers’ QoL (*p* > 0.05).

## 4. Discussion

This study aims to examine the QoL among children with DS based on their age, gender, educational level, school stage, and the influence of their caregivers’ demographic characteristics.

The results of our study show that when we compare children with DS aged 0–2 years with those of other age groups, we notice that the QoL of the former is significantly lower (10.18 ± 3.83). As we can see, the results of our study confirm that children with DS who are less than two years old have a lower QoL than other age groups. This is completely consistent with the study conducted by Scotto and Eymann in Argentina from 2020 to 2021 on children with DS whose ages ranged between two and four years. Their results concur with our study, as they found that these children had a lower QoL score than their unaffected peers. This study was conducted in the psychological, academic (school), and social fields, and the most affected field was the academic (school) field [10]. The American Academy of Pediatrics (AAP) stresses the importance of undergoing all medical evaluations for DS children because they are more susceptible to neurological, heart, and other diseases. Each age group has a set of evaluations, but AAP considers the most important age group to be the early stages, from birth to five years of age [11]. In addition, our study examined the differences between genders in several domains. When we compared females to males, it became clear that females have a lower QoL than males in several domains, including the emotional (*p* = 0.03), social (*p* = 0.01), and school (*p* = 0.01) domains. This finding can be supported by previous research, such as that conducted by Islam et al. on Bangladeshi children with DS. After conducting this study, they found a significant relationship between gender and QoL, particularly in the emotional domain, as females are more concerned with their appearance, and this concern is reflected in their overall QoL [12]. This finding was unsurprising, as caregivers who have worked with DS children have demonstrated that these groups, due to their physical appearance, can experience a decline in their QoL. This hypothesis was aligned with a study conducted in 2017 on children with DS who were experiencing weight-related issues, which in turn affected their QoL [13]. A child’s age and gender certainly influence their QoL. A study conducted by Gaspar et al. showed that adolescents and older children have a lower QoL than younger children due to a greater understanding of the concepts of QoL; this contradicts the results of our study. As for gender, this was consistent with our results, as this study demonstrated that females tend to have a lower QoL in terms of emotional and psychological aspects [14].

Moreover, the most remarkable results across all domains were in the educational level, with uneducated children recording significantly lower QoL scores in the physical (22.34 ± 7.53, *p* = 0.004), emotional (10.41 ± 3.79, *p* = 0.003), social (11.22 ± 4.06, *p* = 0.001), and school (8.75 ± 5.09, *p* = 0.001) domains. The same result was reached in a study conducted by Alrayes et al. in 2024, where they found that education had a positive impact on QoL among DS children, as the educated children showed higher QoL scores [15]. Many studies on people with special needs have shown that regular education has a positive impact on their QoL. The involvement of this group in regular education with normal peers leads to an increase in QoL, which affects various areas of their lives [16].

Our study showed that children with DS who are in primary school (OR = 5.90, 95% CI: 1.85–18.78, *p* = 0.003) and middle school (OR = 5.27, 95% CI: 1.44–19.31, *p* = 0.012) had significantly higher odds of better QoL compared to children with no formal education. A study published in 2013 in the Netherlands examined the impact of educational skills acquired by students with DS from regular education compared to private education. This study found that those children who are continuously exposed to educational materials that are considered a type of regular education have a higher level of mental and cognitive abilities than those in private education, as is positively reflected in their QoL [17].

Additionally, the findings of this study showed that children cared for by their fathers had significantly lower odds of better QoL compared to those cared for by their mothers (OR = 0.07, 95% CI: 0.01–0.90, *p* = 0.04). This hypothesis is consistent with the division imposed by society on parents. The father’s role is usually limited to providing money for his family, whereas the mother has the main role in caring for children. Torquato and his team explained the challenges that fathers face in caring for their children. Fathers, especially those who have DS children, initially struggle to interact with their children, and they often seek to solve their problems using logic. The weak emotional bonds between fathers and their children are also attributed to a lack of social support for these fathers, who are unable to seek help when needed. As a consequence, they feel pressure from society, which negatively impacts their mental health [18]. Many studies have been conducted on the relationship between children with DS and their mothers, and in one of these studies, they concluded that mothers had sufficient ability to help their children to focus while playing, which led to influencing the quality of play and increasing its efficiency. This, in turn, reflects the strong bonds between DS children and their mothers, as the presence of the mother helped to enhance the child’s abilities as a result of his or her feeling of security, which reflects the strong relationship between the child with DS and his or her mother [19].

Our findings in this study showed that none of the demographic characteristics of caregivers reached a sufficient statistical significance level to influence caregivers’ QoL (*p* > 0.05). This result contradicts the results of a study conducted in Jeddah, Saudi Arabia, which found that certain demographic characteristics of parents can positively impact the QoL of children with DS. Among these characteristics are the following: First, family income, as there is a direct relationship between increased income and its impact on children’s physical, psychological, and independence QoL. Second, the educational level of the parents, as the higher the educational level, the more positively impacted the children’s social QoL. On the other hand, some of the results from the Jeddah study were consistent with our results, as other demographic factors such as parents’ age, nationality, or place of residence did not affect QoL [15]. From a scientific perspective, children who are born into low social and economic status will suffer from a negative impact on their QoL level in various areas of life, including physical, emotional, and social levels, as well as school performance. It was found that children who are born into a high social and economic status have the exact opposite. This result suggests that in both cases, the family’s income has an impact on the QoL of the children [20].

In Saudi Arabia, the culture is founded on Islamic principles. A traditional Saudi family is composed of an extended family unit that resides in a single home, including a husband, wife, children, spouses of their children, and grandchildren [21]. Family members in Saudi Arabia accept familial obligations and coexist in harmony, which provides them with a unique identity within society. Consequently, families provide care for a disabled individual rather than an institution [21]. Mothers are frequently the primary caregivers for children with DS in Saudi Arabia as a result of family-oriented norms. As a result, mothers often experience increased emotional stress and heavier responsibilities in the social and psychological domains. The lower QoL level observed among caregivers in our study sample is also likely due to these cultural factors.

## 5. Recommendations for Health Professionals and Practice

A multi-approach strategy is recommended to policymakers in Saudi Arabia: mandatory healthcare training for caregivers and healthcare providers to increase their knowledge concerning DS disabilities and how to support them properly, improved patients’ access to DS-related medications to improve their clinical outcomes, and enhanced development and implementation of anti-discrimination laws for education and healthcare for people with DS. Marshall et al. [22] emphasized the importance of interdisciplinary support for children with DS. They emphasized the necessity of coordinated early care from social workers, psychologists, occupational and developmental therapists, and community facilitators to ensure the holistic well-being of DS people and their caregivers. Caregivers consistently reported that fragmented services impede optimal developmental outcomes, whereas team-based and family-centered approaches simplify access to medical, educational, and psychosocial interventions, thereby promoting social inclusion, emotional resilience, and learning. The integration of these diverse professionals from infancy enhances care coordination and guarantees that support is tailored to changing requirements, thereby significantly contributing to higher QoL levels.

Through the results presented by our study, the importance of accurate and continuous follow-up of DS children has emerged in all areas, whether health, psychological, or physical, considering that the early childhood period is one of the most important stages of their lives that protects them from many future risks. The importance of follow-up lies in visiting clinics and hospitals specializing in children and increasing parents’ awareness of the need to conduct periodic examinations to ensure the health of their children [23]. From a psychological perspective, the concentration of children is high at this stage, as they acquire many skills that remain with them for the rest of their lives. Therefore, a suitable family environment must be created to raise a child with psychological stability [24].

As for the physical aspect, the participation of parents alongside the medical staff is very important to help their children, especially those who experience physical and mental disabilities, in order to increase the children’s self-reliance. Parents can, for example, make an effort to help their children walk or help them in other activities, as this group needs special care [25]. Considering children with DS, whether male or female, it is important to meet all their emotional, physical, social, and academic needs, with a focus on the females, especially in the emotional field, because they are naturally more sensitive to these matters than males. Helping female children with DS involves communicating with them in a positive manner that can be easily understood by a child at a young age. The focus in this dialog is on the importance of self-respect, encouraging pride in any achievement, and giving importance to opinions about clothing or external appearance, for example. It is also necessary to create a personal space for them to express their opinions independently. From a medical standpoint, it is the responsibility of the medical staff to maintain contact with parents to explain any symptoms that their children may experience, as these signs may be related to problems such as anxiety, depression, etc. [26]. Education is considered one of the most important basic elements for any child’s development. For children with DS, being in a natural environment helps them to acquire communication skills with others, which is reflected in their social performance. In addition, continuous commitment to educational materials, like their unaffected peers, plays a role in enhancing their cognitive abilities. Integrating them into regular schools has a positive impact, as has been demonstrated by many previous studies. These children must not be kept among people who share the same syndrome, separately from society. On the contrary, they must be integrated into society. Here, this responsibility falls on parents, teachers in schools, and neighbors at home, as all of these groups must cooperate to enhance their self-confidence [27].

It is important to focus on the importance of parents’ participation in caring for their children with DS, because their participation is considered an important element that reflects on the quality of their lives. Talking to children and sharing with them in various activities increases their self-confidence, which in turn reflects positively on their psychological health. Here, the importance emerges of holding courses and workshops for parents to increase their awareness of the tools and methods of care and the correct understanding of their children’s needs, which helps children grow more effectively and efficiently [28]. Children with DS in the Middle East face many challenges due to the limited number of clinics, hospitals, and care centers for them. Many children are diagnosed late due to insufficient health resources. As for the specialists in the medical team who support DS cases, their numbers are few. In many medical teams, they lack sufficient training in methods of dealing with children with DS, and this, in turn, affects the care provided to them [29,30]. Family income plays a major role in delayed development in children with DS because parents are restricted to themselves as guides to care for their children due to their limited income. Many families experience a lack of specialized care for children with DS in their areas because most of these services are limited to major cities, which makes them difficult to access [15]. From a societal perspective, there is a lack of awareness among members of society about this group due to the lack of campaigns that focus on educating people about how to support these children.

This study has several limitations. The online cross-sectional survey study design has restricted generalizability and the ability to examine causality across the study variables. Online platform users might not fully represent the targeted study population. In addition, self-administered survey studies are prone to reporting bias. Another limitation is the small sample size for certain subgroups, such as the number of families where the father was the caregiver, which might have affected the statistical power for the study findings. Therefore, the study findings should be interpreted carefully. Larger-scale and longitudinal studies are recommended.

## 6. Conclusions

The findings of this study demonstrated a low level of QoL, affecting the emotional, social, and school domains, especially among female children with DS aged between 0 and 2 years with no formal education and cared for by their fathers. Governments should develop a comprehensive plan to care for these children and families to enhance their rights and quality of life, placing emphasis on those who exhibit parameters related to lower QoL. Healthcare providers and caregivers should be required to learn about and address DS issues, patient access to DS medications should be increased to improve clinical outcomes, education and healthcare should be improved, and laws against discrimination should be implemented.

## Figures and Tables

**Table 1:** Demographic and sociodemographic characteristics of children with Down syndrome.

		N	%
Age (years)	0–2	34	14.8
	3–11	79	34.5
	12–14	58	25.3
	15–18	58	25.3
Gender	Female	126	55
	Male	103	45
Residency	Middle	34	14.8
	East	91	39.7
	West	42	18.3
	North	30	13.1
	South	32	14
Education level	None	32	14
	Nursery school	21	9.2
	Kindergarten	29	12.7
	Primary school	77	33.6
	Middle school	38	16.6
	High school	32	14
Working in health sector	No	165	72.1
	Yes	64	27.9
Caregiver	Mother	57	25.7
	Father	8	3.6
	Both	157	70.7
Income (SAR)	Less than 2000	6	2.6
	2001–5000	50	21.8
	5001–10,000	90	39.3
	10,001–20,000	67	29.3
	20,001+	16	7

SAR: Saudi Arabian Riyal.

**Table 2:** Challenges faced by children with Down syndrome in physical, emotional, social, and school activities.

		Never (%)	Rarely (%)	Sometimes (%)	Often (%)	Always (%)
Physical	Walking long distances	43 (18.8)	81 (35.4)	66 (28.8)	32 (14)	7 (3.1)
	Running	32 (14)	52 (22.7)	75 (32.8)	52 (22.7)	18 (7.9)
	Sports activities or exercises	32 (14)	46 (20.1)	80 (34.9)	53 (23.1)	18 (7.9)
	Carrying heavy objects	30 (13.1)	58 (25.3)	64 (27.9)	55 (24)	22 (9.6)
	Bathing or washing independently	69 (30.1)	52 (22.7)	53 (23.1)	34 (14.8)	21 (9.2)
	Household chores	46 (20.1)	63 (27.5)	58 (25.3)	39 (17)	23 (10)
	Experiencing physical pain or aches	48 (21)	79 (34.5)	57 (24.9)	39 (17)	6 (2.6)
	Feeling sluggish or physically fatigued	40 (17.5)	78 (34.1)	73 (31.9)	29 (12.7)	9 (3.9)
Emotional	Feeling afraid	68 (29.7)	69 (30.1)	62 (27.1)	25 (10.9)	5 (2.2)
	Feeling sad	59 (25.8)	70 (30.6)	71 (31)	25 (10.9)	4 (1.7)
	Feeling angry	37 (16.2)	60 (26.2)	67 (29.3)	50 (21.8)	15 (6.6)
	Having trouble sleeping	54 (23.6)	66 (28.8)	58 (25.3)	41 (17.9)	10 (4.4)
	Worrying about the future	93 (40.6)	66 (28.8)	36 (15.7)	26 (11.4)	8 (3.5)
Social	Getting along with peers	39 (17)	69 (30.1)	54 (23.6)	44 (19.2)	23 (10)
	Having trouble making friends	37 (16.2)	70 (30.6)	70 (30.6)	44 (19.2)	8 (3.5)
	Being mocked by peers	79 (34.5)	48 (21)	51 (22.3)	40 (17.5)	11 (4.8)
	Having difficulty doing things that peers do	56 (24.5)	54 (23.6)	63 (27.5)	40 (17.5)	16 (7)
	Having trouble playing with peers	63 (27.5)	61 (26.6)	53 (23.1)	44 (19.2)	8 (3.5)
School	Having difficulty concentrating/paying attention in class	62 (27.1)	70 (30.6)	55 (24)	35 (15.3)	7 (3.1)
	Experiencing forgetfulness	57 (24.9)	51 (22.3)	75 (32.8)	38 (16.6)	8 (3.5)
	Having trouble keeping up with schoolwork	68 (29.7)	48 (21)	62 (27.1)	37 (16.2)	14 (6.1)
	Skipping school due to feeling unwell	74 (32.3)	47 (20.5)	64 (27.9)	36 (15.7)	8 (3.5)
	Skipping school due to hospital visit	54 (23.6)	65 (28.4)	75 (32.8)	24 (10.5)	11 (4.8)

**Table 3:** Scores for physical, emotional, social, and school domains by demographic factors.

		Physical	*p*-Value	Emotional	*p*-Value	Social	*p*-Value	School	*p*-Value
Age (years)	0–2	21.06 ± 8.71	0.79	10.18 ± 3.83	0.02	11.35 ± 4.21	0.13	9.62 ± 5.31	0.004
	3–11	21.52 ± 5.48		12.05 ± 3.35		13.06 ± 3.27		12.59 ± 3.75	
	12–14	21.86 ± 5.33		12.22 ± 3.71		13.14 ± 3.85		12.53 ± 4.16	
	15–18	20.76 ± 6.60		12.52 ± 4.24		12.91 ± 4.39		12.78 ± 4.93	
Gender	Female	20.63 ± 6.09	0.05	11.45 ± 3.97	0.03	12.23 ± 3.98	0.01	11.53 ± 4.39	0.01
	Male	22.21 ± 6.43		12.52 ± 3.53		13.48 ± 3.68		12.98 ± 4.58	
Residency	Middle	21.26 ± 6.81	0.60	11.21 ± 3.65	0.03	11.71 ± 4.09	0.41	11.47 ± 4.78	0.13
	East	21.19 ± 6.17		12.47 ± 3.79		12.88 ± 3.92		12.84 ± 4.27	
	West	20.45 ± 5.61		12.60 ± 3.37		13.45 ± 3.62		12.24 ± 4.00	
	North	22.87 ± 6.79		11.97 ± 3.93		12.90 ± 3.72		12.63 ± 4.90	
	South	21.63 ± 6.47		10.28 ± 4.05		12.72 ± 4.08		10.59 ± 5.01	
Education level	None	22.34 ± 7.53	0.004	10.41 ± 3.79	0.003	11.22 ± 4.06	0.001	8.75 ± 5.09	0.001
	Nursery school	19.19 ± 8.64		11.00 ± 4.14		11.62 ± 4.27		11.14 ± 5.61	
	Kindergarten	20.76 ± 5.52		11.28 ± 3.41		12.86 ± 3.13		11.97 ± 3.89	
	Primary	22.70 ± 5.27		12.94 ± 3.42		13.88 ± 3.16		14.01 ± 3.42	
	Middle	22.24 ± 4.85		13.00 ± 3.55		13.92 ± 3.83		13.29 ± 3.68	
	High school	17.97 ± 6.29		11.00 ± 4.33		11.09 ± 4.60		10.78 ± 4.60	
Working in the health sector	No	21.22 ± 6.38	0.64	11.79 ± 3.83	0.35	12.68 ± 4.02	0.51	12.13 ± 4.69	0.78
	Yes	21.66 ± 6.04		12.31 ± 3.74		13.06 ± 3.55		12.31 ± 4.11	
Care	Mother	21.70 ± 6.60	0.73	11.61 ± 3.50	0.81	13.30 ± 3.78	0.06	12.21 ± 4.67	0.49
	Father	22.13 ± 8.10		11.63 ± 4.17		9.88 ± 4.19		10.25 ± 5.87	
	Both	21.04 ± 6.14		11.97 ± 3.89		12.75 ± 3.87		12.16 ± 4.40	
Income (SAR)	Less than 2000	16.00 ± 7.38	0.007	10.50 ± 6.63	0.18	9.83 ± 2.93	0.03	10.17 ± 5.60	0.05
	2001–5000	21.98 ± 6.25		12.44 ± 3.98		13.56 ± 4.54		13.54 ± 4.95	
	5001–10,000	22.23 ± 5.87		11.91 ± 3.78		13.04 ± 3.65		11.68 ± 4.23	
	10,001–20,000	21.16 ± 6.21		12.18 ± 3.56		12.64 ± 3.51		12.43 ± 4.32	
	20,000+	17.13 ± 6.53		10.00 ± 2.73		10.69 ± 3.98		10.50 ± 4.47	

SAR: Saudi Arabian Riyal.

**Table 4:** Demographic characteristics of the participants.

		N	%
Age (years)	19–25	68	39.8
	26–35	55	32.2
	36–45	24	14
	46–55	16	9.4
	56+	8	4.7
Gender	Female	132	77.2
	Male	39	22.8
Education level	Not educated	5	2.9
	Primary school	15	8.8
	Middle school	37	21.6
	High school	42	24.6
	Bachelor	63	36.8
	Diploma	6	3.5
	Postgraduate	3	1.8
Residence	South	12	7
	East	86	50.3
	North	18	10.5
	West	17	9.9
	Middle	38	22.2
Does either parent work in the healthcare sector?	No	156	91.2
	Yes	15	8.8
Income (SAR)	Less than 2000	8	4.7
	2001–5000	38	22.2
	5001–10,000	59	34.5
	10,001–20,000	48	28.1
	20,001+	18	10.5
Care	Mother	30	17.5
	Father	24	14
	Both	111	64.9
	Others	6	3.5

SAR: Saudi Arabian Riyal.

**Table 5:** The WHOQOL-BREF items with participants’ responses.

	Very poor (%)	Poor (%)	Neither poor nor good (%)	Good (%)	Very good (%)
How would you rate your quality of life?	4 (2.3)	8 (4.7)	34 (19.9)	78 (45.6)	47 (27.5)
	Very dissatisfied (%)	Dissatisfied (%)	Neither satisfied nor dissatisfied (%)	Satisfied (%)	Very satisfied (%)
How satisfied are you with your health?	2 (1.2)	12 (7)	40 (23.4)	71 (41.5)	46 (26.9)
	Not at all (%)	A little (%)	Moderate (%)	Very much (%)	Extremely (%)
To what extent do you feel that physical pain prevents you from doing what you need to do? *	4 (2.3)	10 (5.9)	64 (37.4)	53 (31)	40 (23.4)
How much do you need medical treatment to function in your daily life? *	8 (4.7)	19 (11.1)	49 (28.7)	50 (29.2)	45 (26.3)
How much do you enjoy life?	13 (7.6)	26 (15.2)	58 (33.9)	42 (24.6)	32 (18.7)
To what extent do you feel that your life is meaningful?	19 (11.1)	24 (14)	46 (26.9)	52 (30.4)	30 (17.5)
How well are you able to concentrate?	14 (8.2)	37 (21.6)	60 (35.2)	41 (24)	19 (11.1)
How safe do you feel in your daily life?	10 (5.9)	24 (14)	41 (24)	44 (25.7)	52 (30.4)
How healthy is your physical environment?	20 (11.7)	15 (8.8)	45 (26.3)	56 (32.8)	35 (20.5)
Do you have enough energy for everyday life?	12 (7)	40 (23.4)	50 (29.2)	46 (26.9)	23 (13.5)
Are you able to accept your bodily appearance?	12 (7)	30 (17.5)	53 (31)	38 (22.2)	38 (22.2)
Have you enough money to meet your needs?	14 (8.2)	27 (15.8)	55 (32.2)	45 (26.3)	30 (17.5)
How available to you is the information that you need in your day-to-day life?	19 (11.1)	28 (16.4)	55 (32.2)	41 (24)	28 (16.4)
To what extent do you have the opportunity for leisure activities?	18 (10.5)	39 (22.8)	55 (32.2)	36 (21.1)	23 (13.5)
	Very poor (%)	Poor (%)	Neither poor nor good (%)	Good (%)	Very good (%)
How well are you able to get around?	19 (11.1)	33 (19.3)	51 (29.8)	45 (26.3)	23 (13.5)
	Very dissatisfied (%)	Dissatisfied (%)	Neither satisfied nor dissatisfied (%)	Satisfied (%)	Very satisfied (%)
How satisfied are you with your sleep?	18 (10.5)	21 (12.3)	58 (33.9)	63 (36.8)	11 (6.4)
How satisfied are you with your ability to perform your daily living activities?	11 (6.4)	25 (14.6)	59 (34.5)	56 (32.8)	20 (11.7)
How satisfied are you with your capacity for work?	18 (10.5)	28 (16.4)	55 (32.2)	47 (27.5)	23 (13.5)
How satisfied are you with yourself?	15 (8.8)	21 (12.3)	52 (30.4)	57 (33.3)	26 (15.2)
How satisfied are you with your personal relationships?	15 (8.8)	19 (11.1)	64 (37.4)	48 (28.1)	25 (14.6)
How satisfied are you with your sex life?	18 (10.5)	28 (16.4)	58 (33.9)	48 (28.1)	19 (11.1)
How satisfied are you with the support you receive from your friends?	12 (7)	23 (13.5)	55 (32.2)	53 (31)	28 (16.4)
How satisfied are you with the conditions of your living place?	15 (8.8)	26 (15.2)	47 (27.5)	55 (32.2)	28 (16.4)
How satisfied are you with your access to health services?	9 (5.3)	18 (10.5)	60 (35.1)	53 (31)	31 (18.1)
How satisfied are you with your mode of transportation?	12 (7)	29 (17)	50 (29.2)	53 (31)	27 (15.8)
How often do you have negative feelings, such as blue mood, despair, anxiety, and depression? *	12 (7)	47 (27.5)	55 (32.2)	40 (23.4)	17 (9.9)

* Reverse-coded items.

**Table 6:** Scores for physical, psychological, social, and school domains by demographic factors.

		Physical	*p*-Value	Psychological	*p*-Value	Social Relationships	*p*-Value	Environment	*p*-Value
Age (years)	19–25	59.87 ± 16.28	0.16	57.48 ± 18.93	0.99	57.72 ± 23.67	0.52	62.13 ± 21.46	0.33
	26–35	57.47 ± 17.57		55.08 ± 18.78		52.88 ± 23.36		55.57 ± 20.94	
	36–45	51.04 ± 14.61		51.56 ± 14.16		51.04 ± 22.02		51.95 ± 19.89	
	46–55	60.94 ± 18.56		60.68 ± 21.62		71.35 ± 25.63		63.87 ± 21.53	
	56+	56.70 ± 20.33		50.00 ± 20.65		57.29 ± 22.02		51.17 ± 22.96	
Gender	Female	59.33 ± 16.27	0.74	56.22 ± 18.69	0.47	57.89 ± 23.37	0.45	59.66 ± 20.77	0.66
	Male	52.66 ± 18.46		54.49 ± 18.59		51.71 ± 25.01		53.45 ± 22.87	
Residency	South	53.57 ± 14.61	0.00	56.94 ± 15.01	0.00	49.31 ± 16.84	0.00	59.90 ± 18.12	0.001
	East	59.97 ± 18.20		56.15 ± 20.44		58.82 ± 25.70		59.01 ± 22.74	
	North	51.19 ± 16.25		51.62 ± 13.42		52.31 ± 22.83		51.04 ± 21.52	
	West	59.03 ± 15.78		58.58 ± 20.12		57.84 ± 20.51		61.03 ± 24.36	
	Middle	56.86 ± 15.16		55.48 ± 17.22		54.82 ± 23.30		58.14 ± 17.63	
Does either parent work in the healthcare sector?	No	58.10 ± 17.12	0.10	56.14 ± 18.57	0.58	57.16 ± 23.97	0.27	58.91 ± 21.27	0.85
	Yes	54.76 ± 15.49		52.50 ± 19.53		49.44 ± 21.70		51.25 ± 21.74	
Income (SAR)	20,001+	66.27 ± 16.40	0.04	53.94 ± 22.48	0.69	65.28 ± 22.37	0.27	64.24 ± 22.64	0.29
	Less than 2000	61.61 ± 12.48		55.73 ± 22.81		61.46 ± 20.38		52.34 ± 14.25	
	10,001–20,000	57.96 ± 16.28		56.16 ± 18.43		58.16 ± 23.29		58.01 ± 22.95	
	2001–5000	54.51 ± 16.95		53.18 ± 16.71		51.10 ± 25.05		56.33 ± 20.38	
	5001–10,000	56.72 ± 17.77		57.84 ± 18.51		55.23 ± 24.02		58.63 ± 21.28	
Education level	Not educated	40.71 ± 10.29	0.001	52.50 ± 3.73	0.21	58.33 ± 13.18	0.05	52.50 ± 9.48	0.01
	Primary school	54.05 ± 15.85		53.33 ± 15.69		52.78 ± 15.96		56.88 ± 19.27	
	Middle school	50.10 ± 17.18		53.38 ± 18.29		47.30 ± 27.43		51.18 ± 23.24	
	High school	57.99 ± 14.02		51.49 ± 19.15		55.16 ± 18.49		53.87 ± 17.93	
	Bachelor	62.64 ± 16.53		59.99 ± 19.23		61.38 ± 25.76		64.19 ± 21.79	
	Diploma	70.24 ± 18.85		59.72 ± 19.84		73.61 ± 22.62		69.27 ± 20.86	
	Postgraduate	71.43 ± 19.88		69.44 ± 17.35		66.67 ± 14.43		76.04 ± 22.61	
Care	Mother	54.76 ± 13.57	0.24	52.22 ± 14.34	0.13	56.11 ± 19.69	0.66	55.83 ± 18.73	0.20
	Father	52.98 ± 17.77		51.04 ± 16.95		54.51 ± 28.34		52.47 ± 22.89	
	Both	59.59 ± 17.15		57.21 ± 19.67		56.38 ± 23.76		59.46 ± 21.48	
	Others	59.52 ± 23.55		67.36 ± 19.44		68.06 ± 27.60		70.83 ± 22.42	

SAR: Saudi Arabian Riyal.

**Table 7:** Factors associated with quality of life in children with Down syndrome.

		AOR (95% CI)	*p*-Value
Age (years)	0–2	Reference	
	3–11	0.62 (0.19–1.95)	0.410
	12–14	1.21 (0.35–4.14)	0.766
	15–18	1.80 (0.50–6.49)	0.371
Gender	Female	Reference	
	Male	1.85 (0.96–3.58)	0.067
Residency	Middle	Reference	
	East	1.93 (0.70–5.28)	0.201
	West	1.59 (0.53–4.77)	0.407
	North	2.40 (0.71–8.06)	0.157
	South	1.44 (0.44–4.74)	0.552
Education level	None	Reference	
	Nursery school	1.20 (0.31–4.71)	0.793
	Kindergarten	2.67 (0.72–9.94)	0.143
	Primary	5.90 (1.85–18.78)	0.003
	Middle	5.27 (1.44–19.31)	0.012
	High school	1.05 (0.27–4.14)	0.947
Working in the health sector	No	Reference	
	Yes	1.72 (0.80–3.68)	0.163
care	Mother	Reference	
	Father	0.07 (0.01–0.90)	0.041
	Both	0.76 (0.37–1.56)	0.456
Income (SAR)	Less than 2000	Reference	
	2001–5000	2.80 (0.34–22.99)	0.339
	5001–10,000	2.24 (0.28–17.88)	0.445
	10,001–20,000	2.57 (0.31–21.05)	0.380
	20,001+	0.90 (0.08–10.27)	0.934
	Constant	0.10 (0.00–0.00)	0.062

SAR: Saudi Arabian Riyal.

**Table 8:** Factors associated with quality of life among caregivers.

		AOR (95% CI)	*p*-Value
Age (years)	19–25	Reference	
	26–35	1.44 (0.64–3.25)	0.379
	36–45	0.84 (0.29–2.45)	0.748
	46–55	1.55 (0.40–5.95)	0.525
	56+	3.72 (0.57–24.26)	0.170
Gender	Female	Reference	
	Male	1.50 (0.58–3.88)	0.408
Residency	South	Reference	
	East	1.08 (0.44–2.64)	0.873
	West	2.12 (0.55–8.15)	0.274
	North	0.50 (0.12–2.15)	0.351
	Middle	0.27 (0.05–1.55)	0.141
Education level	None	Reference	
	Primary	1.81 (0.17–19.74)	0.627
	Middle	0.92 (0.09–9.20)	0.942
	High school	0.86 (0.08–9.20)	0.902
	Bachelor	0.62 (0.06–6.74)	0.694
	Diploma	0.23 (0.01–4.50)	0.336
	Postgraduate	0.53 (0.01–20.53)	0.735
Working in the healthcare sector	No	Reference	
	Yes	0.55 (0.15–2.10)	0.384
Care	Mother	Reference	
	Father	0.54 (0.15–1.90)	0.334
	Both	1.04 (0.41–2.64)	0.934
	Other	1.24 (0.15–10.26)	0.841
Income (SAR)	Less than 2000	Reference	
	2001–5000	1.25 (0.18–8.95)	0.821
	5001–10,000	0.76 (0.11–5.12)	0.775
	10,001–20,000	1.05 (0.15–7.19)	0.958
	20,001+	1.41 (0.16–12.34)	0.755
	Constant	1.13 (0.00–0.00)	0.938

SAR: Saudi Arabian Riyal.

## Data Availability

Data is contained within the article.
